# Interleukin-13-Overexpressing Mice Represent an Advanced Preclinical Model for Detecting the Distribution of Antimycobacterial Drugs within Centrally Necrotizing Granulomas

**DOI:** 10.1128/aac.01588-21

**Published:** 2022-05-23

**Authors:** Kerstin Walter, Julia Kokesch-Himmelreich, Axel Treu, Franziska Waldow, Doris Hillemann, Nikolas Jakobs, Ann-Kathrin Lemm, Dominik Schwudke, Andreas Römpp, Christoph Hölscher

**Affiliations:** a Division of Infection Immunology, Research Center Borstel, Leibniz Lung Center, Borstel, Germany; b German Center for Infection Research (DZIF), Thematic Translational Unit Tuberculosis, Braunschweig, Germany; c Chair of Bioanalytical Sciences and Food Analysis, University of Bayreuth, Bayreuth, Germany; d Division of Bioanalytical Chemistry, Research Center Borstel, Leibniz Lung Center, Borstel, Germany; e National Reference Center for Mycobacteria, Research Center Borstel, Leibniz Lung Center, Borstel, Germany; f German Center for Lung Research (DZL), Airway Research Center North (ARCN), Research Center Borstel, Leibniz Lung Center, Borstel, Germany

**Keywords:** antibiotics, murine models, pathology, tuberculosis

## Abstract

The Mycobacterium tuberculosis-harboring granuloma with a necrotic center surrounded by a fibrous capsule is the hallmark of tuberculosis (TB). For a successful treatment, antibiotics need to penetrate these complex structures to reach their bacterial targets. Hence, animal models reflecting the pulmonary pathology of TB patients are of particular importance to improve the preclinical validation of novel drug candidates. M. tuberculosis-infected interleukin-13-overexpressing (IL-13^tg^) mice develop a TB pathology very similar to patients and, in contrast to other mouse models, also share pathogenetic mechanisms. Accordingly, IL-13^tg^ animals represent an ideal model for analyzing the penetration of novel anti-TB drugs into various compartments of necrotic granulomas by matrix-assisted laser desorption/ionization–mass spectrometry imaging (MALDI-MS imaging). In the present study, we evaluated the suitability of BALB/c IL-13^tg^ mice for determining the antibiotic distribution within necrotizing lesions. To this end, we established a workflow based on the inactivation of M. tuberculosis by gamma irradiation while preserving lung tissue integrity and drug distribution, which is essential for correlating drug penetration with lesion pathology. MALDI-MS imaging analysis of clofazimine, pyrazinamide, and rifampicin revealed a drug-specific distribution within different lesion types, including cellular granulomas, developing in BALB/c wild-type mice, and necrotic granulomas in BALB/c IL-13^tg^ animals, emphasizing the necessity of preclinical models reflecting human pathology. Most importantly, our study demonstrates that BALB/c IL-13^tg^ mice recapitulate the penetration of antibiotics into human lesions. Therefore, our workflow in combination with the IL-13^tg^ mouse model provides an improved and accelerated evaluation of novel anti-TB drugs and new regimens in the preclinical stage.

## INTRODUCTION

Worldwide, around 10 million people were newly diagnosed with tuberculosis (TB) in 2019 and around 1.5 million died from this disease (1). The treatment of TB is becoming more and more challenging due to the increasing development of multidrug-resistant (MDR) and extensively drug-resistant (XDR) Mycobacterium tuberculosis strains, particularly in Eastern Europe, sub-Saharan Africa, and Asia. Hence, only novel treatment strategies will allow a significant reduction of TB incidence, for both resistant and drug-sensitive cases, and these strategies demand the discovery of new drugs (2).

Whereas most other bacterial infections are cured by a monotherapy in a very short period of time, the treatment of drug-sensitive pulmonary TB with multiple antibiotics usually takes 6 months (1). This long-lasting multidrug therapy often hampers compliance and adherence to medication instructions, leading to drug failure, reactivation, and generation of drug-resistant bacteria. One explanation for the long duration of therapy for TB is the granulomatous lung pathology of this chronic infectious disease (3). In TB patients, pulmonary lesions develop from initially cellular to necrotizing granulomas (4–6). The necrotic center is surrounded by a layer of foamy macrophages and a collagen capsule that walls off the granuloma from the adjoining tissue. In cellular granulomas, M. tuberculosis mostly resides within macrophages. In necrotic granulomas, however, the center often harbors large numbers of extracellular bacilli. Hence, to successfully treat pulmonary TB, antibiotics have to breach the fibrous cuff and permeate these complex lesions in order to reach their mycobacterial targets.

However, most anti-TB therapeutics have been introduced into the clinic without considering the dynamic distribution within granulomas (6). Former studies already pointed out that the concentrations of the first-line drugs isoniazid, rifampicin (RIF), and pyrazinamide (PZA) are lower within pulmonary lesions than concentrations in plasma (3, 7). With its unique combination of molecular and spatial information, matrix-assisted laser desorption/ionization–mass spectrometry (MALDI-MS) imaging is an ideal tool to visualize the spatial distribution of antibiotics in lung tissue and necrotic granulomas, including sublesional compartments. It allows the identification and differentiation of multiple compounds without radioactive labeling in a single measurement (8, 9). Here, a laser beam is deployed to scan the matrix-coated tissue section. The molecules are desorbed and ionized in the laser focus, and a mass spectrum is acquired from every spot of the measurement area. MALDI-MS imaging was successfully used to investigate the distributions of a number of drug compounds in thin tissue sections (10, 11), and we have previously developed a MALDI-MS imaging workflow for the detection of anti-TB drugs in mouse lung tissue (12). MALDI-MS imaging has also been applied to examine the distributions of various TB antibiotics in pulmonary granulomas of M. tuberculosis-infected rabbits, such as moxifloxacin, clofazimine (CFZ), PZA, and RIF (13–16), revealing that the antibiotic distribution is often drug specific and depends on the lesion type. From these studies, it can be concluded that the concentrations of some anti-TB drugs in necrotic granulomas are often insufficient (6). Especially, the necrotic lesion center, which often harbors large numbers of extracellular, slowly replicating or nonreplicating bacilli, is a compartment that is difficult to reach with drugs. Therefore, bacteria in this niche are most likely to acquire a “persister” phenotype, with slow metabolism and tolerance against high drug concentrations, making effective therapy even more challenging. As a consequence, insufficient concentrations of some drugs especially in the necrotic center seem to foster the development of resistance (17). Together, in pharmacokinetic (PK) and pharmacodynamic (PD) preclinical studies, the complex structure of necrotizing granulomas in pulmonary TB lesions has to be considered for a comprehensive evaluation of novel and effective antimycobacterial therapeutics.

The standard mouse model, which is mostly used for preclinical anti-TB drug and regimen evaluation, shows after infection with M. tuberculosis only cellular and not centrally necrotizing granulomas (18). However, the C3HeB/FeJ substrain of the inbred mouse strain C3H has been shown to develop necrotic pulmonary lesions after infection with M. tuberculosis (19). Using a hypothesis-driven approach based on findings on the pathogenesis of human TB, we have previously developed a different mouse model also showing human-like granulomatous lesions (18). In human TB, an increased production of the cytokines interleukin (IL)-4 and IL-13 is associated with lung damage (20, 21). Since no functional connection between IL-4/IL-13 and the development of granuloma necrosis in TB patients was available, we previously analyzed the association of gene variants in the common receptor subunit IL4RA and disease severity in a large patient TB cohort. We revealed that a structural variant of IL4RA known to enhance signal transduction by IL-4/IL-13 was significantly linked to greater cavity size (22). Our principal finding that increased IL-4 receptor alpha-mediated signaling is linked to TB pathogenesis in humans was recently corroborated by a meta-analysis of the association between IL-4 rs2243250 polymorphism and the likelihood of developing TB (23). Because IL-4 and IL-13 are hardly expressed after M. tuberculosis infection in wild-type mice, which do not form centrally necrotizing granulomas, we hypothesized that an increase in IL-13 expression leads to the development of necrotic lesions. In fact, M. tuberculosis infection of IL-13-overexpressing (IL-13^tg^) mice results in lesions strongly resembling the pathology of human TB: central necrosis with a strict stratification of a fibrous capsule that separates the necrotizing granuloma from the adjoining tissue, foamy macrophages, found adjacent to the fibrous rim within the necrotic core, and dispersed multinucleated giant cells (18, 24, 25).

Hence, as M. tuberculosis-infected IL-13^tg^ mice not only recapitulate human TB pathology but also share pathogenetic mechanisms (22), these animals are an ideal preclinical model for the evaluation of new antimycobacterial drugs and, in particular, for analyzing the distribution of these therapeutics into sublesional compartments of necrotic granulomas by MALDI-MS imaging. For this purpose, we, at the Research Center Borstel and the University of Bayreuth, have combined our expertise in experimental TB, bioanalytical chemistry and MALDI-MS imaging in a preclinical platform of the Thematic Translational Unit–Tuberculosis (TTU-TB) at the German Center for Infection Research (DZIF). To validate IL-13^tg^ mice as an appropriate model for determining the antibiotic distribution within granulomatous lesions, we infected these animals with M. tuberculosis, treated them with PZA, CFZ, and RIF, antibiotics whose penetration into the centrally necrotizing granulomas is known (17), and measured the drug distribution by an optimized MALDI-MS imaging method (12). While the present manuscript focuses on the validation of the preclinical IL-13^tg^ model and the inactivation of M. tuberculosis for subsequent drug detection, we describe in a separate analytical study the MALDI-MS imaging acquisition and data analysis in further detail (82).

## RESULTS

### Histopathology in BALB/c IL-13^tg^ mice at the onset of drug therapy.

**FIG 1:**
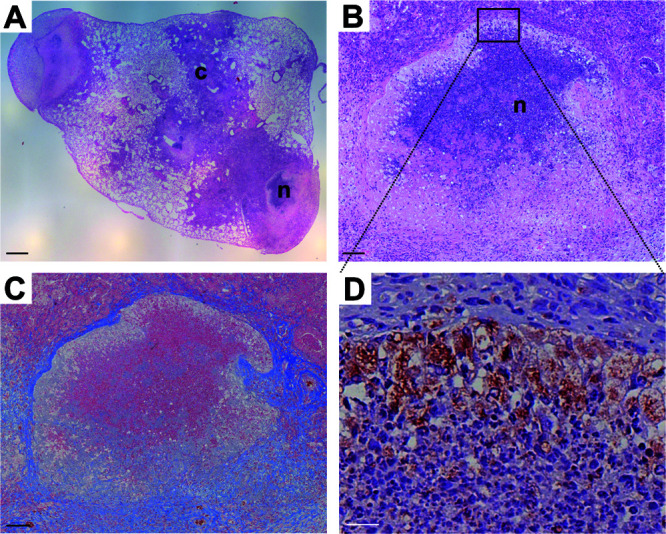
BALB/c IL-13^tg^ mice develop a human-like pathology after M. tuberculosis infection. BALB/c IL-13^tg^ mice were infected with 263 CFU of M. tuberculosis H37Rv via the aerosol route. After 9 weeks, sections from formalin-fixed and paraffin-embedded lungs were prepared and subjected to histopathological and immunohistochemical analysis. (A) HE staining revealed multiple lesion types ranging from cellular (c) to centrally necrotizing granulomas (n). (B) Higher-magnification image of panel A illustrating the centrally necrotizing granuloma, which is surrounded by a fibrous cuff detected by trichrome staining (C) and a rim of macrophages detected by CD68 staining (D). Representative photomicrographs of 4 mice are shown. Scale bars, 500 μm (A), 100 μm (B and C), and 20 μm (D).

At late time points after aerosol infection with M. tuberculosis H37Rv, BALB/c IL-13^tg^ mice develop necrotic encapsulated granulomas (18), which are widely observed in human TB. Therefore, an optimal utilization of this mouse model for the correlation of lesion-specific pathology and drug penetration measured by MALDI-MS imaging requires sufficient time for necrotic pathology to develop. To assess the formation of granulomas after aerosol infection, lung tissue sections were histologically examined by 9 weeks postinfection. At this time point, an extensive pulmonary inflammation with cellular granulomas but also centrally necrotizing granulomas was observed (Fig. 1A). Cellular, inflammatory lesions were composed primarily of macrophages, lymphocytes, and neutrophils. For centrally necrotizing granulomas, a stringent stratification was evident, with an eosinophilic necrotic core consisting of dead and dying cells that was adjacent to regions of neutrophils (Fig. 1B and D), surrounded by a rim of foamy macrophages staining less intensely with eosin and positive for CD68 (Fig. 1D). A fibrous capsule demarcated the necrotic area from the adjoining tissue (Fig. 1C). Since the development of centrally necrotizing granulomas resembling the pathology of human TB was confirmed, drug treatment of BALB/c IL-13^tg^ mice started 9 weeks after aerosol infection.

### Workflow for drug detection in lesions of BALB/c IL-13^tg^ mice.

**FIG 2:**
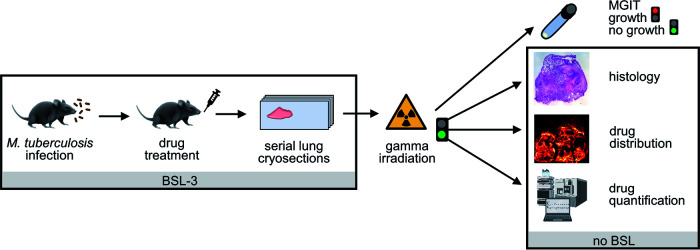
Workflow for drug detection in pulmonary lesions of BALB/c IL-13^tg^ mice. Infection of BALB/c IL-13^tg^ mice with M. tuberculosis H37Rv, drug treatment after necrotizing granulomas have developed, and lung tissue preparation were performed in a BSL-3 laboratory. Inactivation of pulmonary M. tuberculosis by gamma irradiation was confirmed by the MGIT growth system before consecutive cryosections were processed for subsequent analyses, including histological characterization, drug distribution by MALDI-MS imaging, and drug quantification by LC-MS/MS (LC-MS/MS image created using Biorender).

To subsequently validate BALB/c IL-13^tg^ mice as an improved preclinical model for drug penetration studies into TB lesions, a workflow for sample preparation and analysis that considers safety regulations and provides drug distribution data had to be established (Fig. 2). To this end, M. tuberculosis-infected BALB/c IL-13^tg^ mice containing centrally necrotizing granulomas were treated with the drug combination of CFZ, PZA, and RIF, since the penetration of these drugs into human necrotic granulomas has been previously described by Prideaux et al. (17). Following therapy for a total of 10 consecutive days, mice were sacrificed and lung tissue was collected for later serial cryosections, whereby all work was conducted under biosafety level 3 (BSL-3) conditions. Analytical data are generally acquired in chemical laboratories (liquid chromatography-tandem MS [LC-MS/MS], MALDI-MS imaging) operating without biosafety level. Therefore, the inactivation of M. tuberculosis by gamma irradiation and its confirmation using the mycobacterial growth indicator tube (MGIT) system (26) were established. Only samples with associated culture-negative results were further processed. Also, the impact of the identified irradiation dose on tissue morphology, drug concentration, and drug distribution was investigated. The analysis of gamma-irradiated serial cryosections finally allowed a correlation of lung histopathology, drug distribution, and drug quantification.

### Inactivation of M. tuberculosis within centrally necrotizing granulomas by gamma irradiation.

Since previous studies applied gamma irradiation for the inactivation of mycobacteria prior to drug detection by LC-MS/MS or MALDI-MS imaging measurements (7, 13), this method was also used in the present study. In a first approach, frozen lung tissue biopsy specimens containing granulomatous lesions were exposed to different doses of gamma irradiation while frozen on dry ice, as radiation-induced damage of large molecules in hydrated materials is reduced at low temperatures (27). By applying a fixed position for the ^137^Cs irradiation source and a rotation of the samples, an energy dose rate of 11.2 Gy/min was obtained. Subsequently, irradiated tissue was homogenized and inactivation of M. tuberculosis was evaluated by growth in liquid culture using the MGIT system, followed by detection of acid-fast bacilli (AFB) and cord formation in cultures. For homogenate generated from tissue that was not irradiated, a mean time to liquid culture positivity (TTP) of 4 days was determined (Table 1). Gamma irradiation of sample biopsy specimens with 16.10 or 32.20 kGy reduced the amount of viable M. tuberculosis, and the average time to positivity was 22 days or 19 days, respectively. However, even irradiation with 48.30 kGy did not completely eliminate M. tuberculosis, and in 2 out of 3 irradiated biopsy specimens, growth was detected, with a TTP value of 16 or 39 days. Since gamma irradiation of lung tissue biopsy specimens did not completely eradicate M. tuberculosis, in a second approach, cryosections were prepared and irradiated. To this end, lung tissue was cut into 12-μm-thick cryosections, which were mounted onto adhesive microscope slides and frozen at −80°C. During irradiation, sections were kept frozen on dry ice and a homogenous dose distribution was achieved by selecting an irradiation program with an oscillating irradiation source combined with a rotation of the specimens, resulting in an energy dose rate of 4.1 Gy/min. Subsequently, the sections were transferred from the microscope slides into liquid medium, and growth of M. tuberculosis was monitored using the MGIT system. For sections that were not irradiated, the mean TTP value was 14 days (Table 2). Irradiation of cryosections with 0.24 kGy, 0.49 kGy, or 0.98 kGy reduced the amount of viable M. tuberculosis so that a prolonged TTP was observed. After irradiation with 1.95 kGy, 3 out of 4 samples were MGIT negative and negative for AFB and only one sample was still TTP positive (18 days). Irradiation of cryosections with doses of 5.85 kGy, 11.71 kGy, or 17.56 kGy completely inactivated M. tuberculosis so that all MGIT tubes remained negative until day 84 of incubation and also no AFB were detected. Consequently, we identified that 5.85 kGy of gamma irradiation of cryosections is the lowest dose to inactivate M. tuberculosis H37Rv within centrally necrotizing granulomas of BALB/c IL-13^tg^ mice.

**TABLE 1:** Inactivation of M. tuberculosis H37Rv in centrally necrotizing granulomas by gamma irradiation of lung tissue biopsy specimens from M. tuberculosis-infected BALB/c IL-13^tg^ mice^*a*^

Exposed dose (kGy)	TTP (days)	AFB, cord formation^*b*^
None	4 ± 0.5	+
16.10	22 ± 11.0	+
32.20	19 ± 5.4	+
48.30	28 ± 16.2	+

a BALB/c IL-13^tg^ mice were infected with 70 CFU of M. tuberculosis H37Rv, and at time points later than 100 days after infection, lung tissue biopsy specimens were prepared. At least 3 biopsy specimens were gamma irradiated at the indicated doses. The tissue was homogenized after irradiation, and the homogenate was split into 4 aliquots prior to inoculation of MGIT tubes. Inactivation of M. tuberculosis was evaluated by determining the TTP using the MGIT system, followed by microscopic detection of AFB and cord formation in cultures. TTP data are the mean ± SD.

b +, positive for AFB and cord formation.

**TABLE 2:** Inactivation of M. tuberculosis H37Rv in centrally necrotizing granulomas by gamma irradiation of lung cryosections from M. tuberculosis-infected BALB/c IL-13^tg^ mice^*a*^

Exposed dose (kGy)	TTP (days)	AFB, cord formation^*b*^
None	14 ± 1.3	+
0.24	16 ± 0.3	+
0.49	18 ± 1.2	+
0.98	17 ± 1.3	+
1.95	18^*c*^	+
5.85^*d*^	—^*e*^	—
11.71	—	—
17.56	—	—

a BALB/c IL-13^tg^ mice were infected with 70 CFU of M. tuberculosis H37Rv, and at time points later than 100 days after infection, lung tissue biopsy specimens were isolated for subsequent preparation of cryosections. At least 3 slides, each containing 3 cryosections, were gamma irradiated at the indicated doses. After irradiation, sections were detached from the glass slides, whereby cryosections of each slide were pooled and homogenized in sterile water for subsequent inoculation of MGIT tubes. Inactivation of M. tuberculosis was evaluated by determining the TTP using the MGIT system, followed by microscopic detection of AFB and cord formation in cultures. TTP data are the mean ± SD.

b +, positive for AFB and cord formation.

c Irradiation eliminated M. tuberculosis in 3 out of 4 samples.

d Inactivation of M. tuberculosis was confirmed in 3 repeated experiments.

e —, no mycobacterial growth or AFB.

### Effect of irradiation on tissue morphology, drug concentration, and distribution.

**FIG 3:**
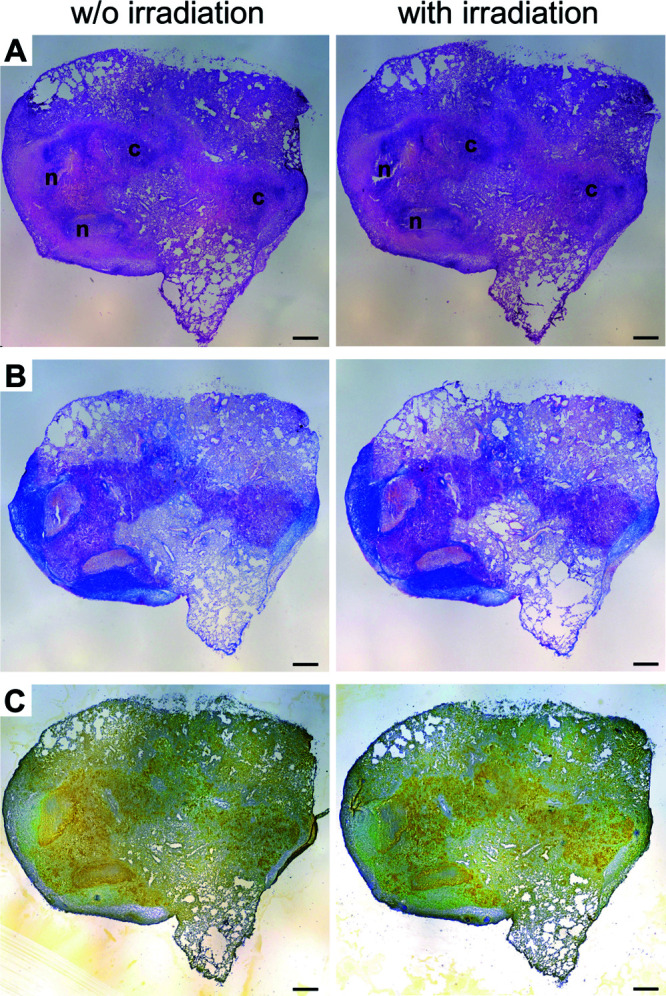
Effect of cryosection irradiation on tissue morphology. BALB/c IL-13^tg^ mice were infected with 263 CFU of M. tuberculosis H37Rv, and after 11 weeks, frozen lung tissue biopsy specimens were prepared and further processed. Serial cryosections were cut, and adjacent sections were either irradiated or not irradiated. Subsequent histological evaluation by HE (A), trichrome (B), or CD68/ZN (C) staining revealed cellular granulomas (c) and necrotic granulomas (n) surrounded by a fibrous cuff. Photomicrographs are representative for two irradiation runs, followed by the respective stainings. Scale bar, 500 μm.

In order to investigate if gamma irradiation of cryosections has an effect on tissue morphology, serial cryosections from M. tuberculosis-infected BALB/c IL-13^tg^ mice were prepared, followed by irradiation of every other section with the dose of 5.85 kGy on dry ice, while the adjacent control sections were processed identically except for the irradiation procedure (see Fig. S1 in the supplemental material for a detailed description of sample preparation). Tissue integrity was assessed by histological and immunohistochemical staining (Fig. 3). Hematoxylin-eosin (HE) staining of irradiated or nonirradiated consecutive sections revealed no differences regarding the morphology of necrotic (Fig. 3A, n) and cellular regions of granulomas (Fig. 3A, c) or uninvolved tissue. Also, the fibrous capsule which demarcates the necrotic granuloma from the adjoining lung tissue was not altered after irradiation (Fig. 3B). Furthermore, immunohistochemical staining of CD68 showed that detection of CD68-expressing macrophages was similar in irradiated and nonirradiated adjacent cryosections, with macrophages located in cellular regions of granulomas as well as next to the fibrous rim of necrotizing granulomas (Fig. 3C), demonstrating that irradiation has no major impact on the integrity of cells such as macrophages.

**FIG 4:**
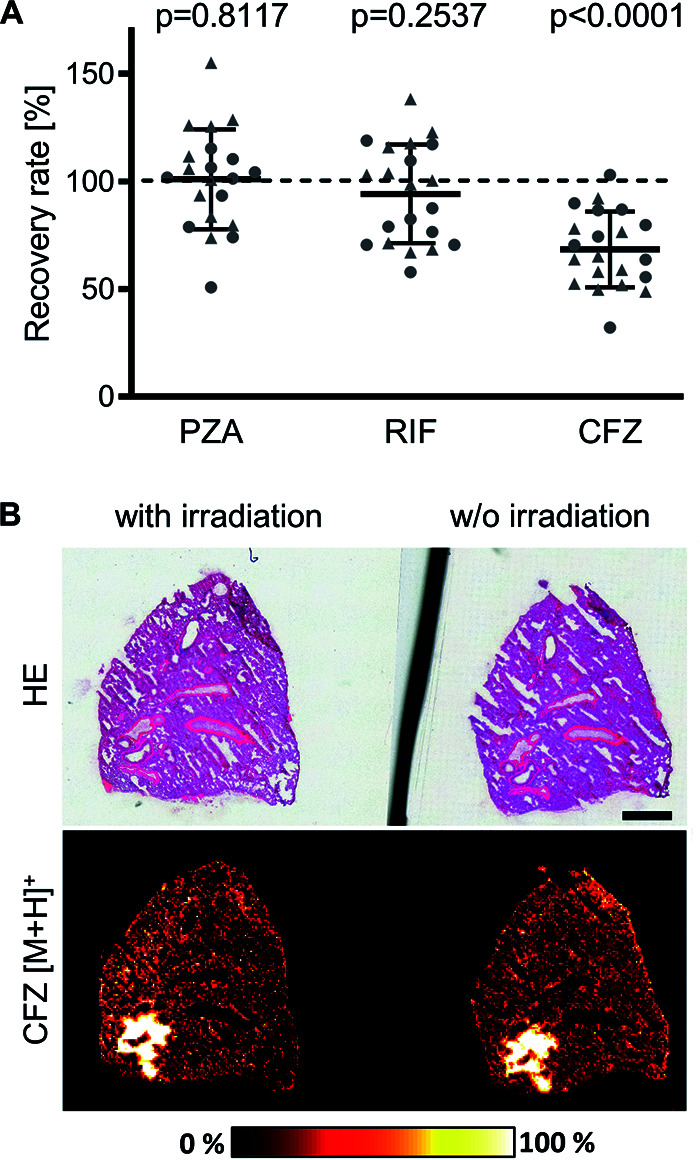
Influence of cryosection irradiation on drug concentrations and distribution. Serial cryosections were prepared from lungs of CFZ/PZA/RIF-treated, naive BALB/c mice for subsequent irradiation of every second section. (A) Recovery rates for PZA, RIF, or CFZ were determined in 2 animals (solid triangle or solid circle). For each mouse, at least 20 consecutive cryosections were prepared for LC-MS/MS measurements after irradiation of every other section. To determine the drug recovery rates, the mean concentrations in nonirradiated cryosections were calculated for each drug, which were subsequently related to the concentrations measured in individual irradiated cryosections (Table S4). Data for independent irradiation experiments with LC-MS/MS quantification are shown as the mean ± SD (*n* = 10 or 11). Statistical analysis was performed by the D’Agostino-Pearson normality test and the Wilcoxon signed rank test. (B) Influence of gamma irradiation on the distribution of CFZ. HE staining of an irradiated cryosection is shown on the left, and the nonirradiated adjacent cryosection on the right. For a direct comparison of the drug distribution in both sections, MALDI-MS imaging of CFZ [M+H]^+^ (*m/z* 473.12942) was performed on both sections simultaneously and in one measurement with a pixel size of 35 μm using DHAP matrix. Scale bar, 1 mm.

In addition, the influence of gamma irradiation on the concentration of drugs within cryosections was investigated. For this purpose, naive BALB/c mice were treated with the CFZ/PZA/RIF combination for 5 consecutive days, and lung lobes were isolated and cut into 12-μm-thick serial cryosections. Frozen sections were irradiated (5.85 kGy) on dry ice, while the neighboring control sections were kept on dry ice without irradiation. In all sections, drug concentrations were quantified by LC-MS/MS analysis (see Fig. S1 for a detailed description of sample processing). The effect of gamma irradiation on the drug concentrations was determined by calculating the recovery rate for CFZ, PZA, and RIF in those sections that were irradiated in comparison to neighboring sections that were not irradiated (Fig. 4A). Concentrations of PZA were similar in cryosections irrespective of irradiation (Table S4), resulting in a mean recovery rate of 101% (standard deviation [SD], 23.17%), while the mean recovery rate of RIF within irradiated sections was slightly reduced to 94% (SD, 22.89%). Concentrations of CFZ were lower after irradiation with 5.85 kGy than in nonirradiated samples (Table S4), so that the mean recovery rate of CFZ was significantly reduced to 68% (SD, 17.63%) in irradiated cryosections. Since the concentration of CFZ was reduced after irradiation, we next investigated whether the distribution of CFZ is altered due to irradiation. To this end, drug distribution was analyzed by MALDI-MS imaging in adjacent lung cryosections that were either irradiated or not irradiated (see Fig. S1 for a detailed description of sample processing). Under both conditions, CFZ penetrated homogeneously throughout the healthy lung parenchyma and higher intensities were observed in close proximity to major airways that were distinguished by subsequent HE staining, demonstrating that irradiation has no impact on the distribution of CFZ (Fig. 4B).

Taking these findings together, we identified that an irradiation dose of 5.85 kGy eliminates M. tuberculosis within cryosections but affects neither tissue morphology nor the cellular composition of sections. Furthermore, gamma irradiation has no obvious impact on the concentrations of PZA and RIF. While the concentration of CFZ was reduced, the distribution of CFZ was not altered due to irradiation. Consequently, we successfully established a workflow for M. tuberculosis inactivation while preserving tissue morphology and staining properties, which are prerequisites for the detection of drug distribution by MALDI-MS imaging in conjunction with lesion-specific pathology.

### Drug distribution in centrally necrotizing and cellular lesions.

**FIG 5:**
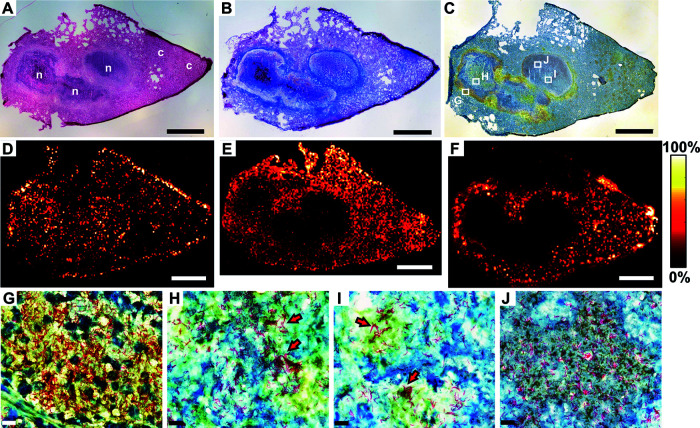
Distribution of CFZ, RIF, and PZA in centrally necrotizing granulomas of BALB/c IL-13^tg^ mice. BALB/c IL-13^tg^ mice were infected with 263 CFU of M. tuberculosis H37Rv. After 9 weeks, animals were treated with CFZ/PZA/RIF for 10 days, and 1 h after the last administration, lung tissue was collected and serial cryosections were prepared for histological characterization of lesions and MALDI-MS imaging analysis. Correlation of lesion pathology (A to C; G to J) and drug distribution (D to F) in neighboring lung cryosections of a BALB/c IL-13^tg^ mouse are shown. (A) HE staining revealed cellular, inflammatory lesions (c) and highly organized centrally necrotizing granulomas (n), which are surrounded by a rim of macrophages (C) detected by CD68/ZN staining and encapsulated by a fibrous cuff (B) detected by trichrome staining. (D) Distribution of PZA [M + 2H]^+·^ (*m/z* 125.05836), pixel size of 35 μm, DHB matrix. (E) Distribution of RIF [M-H]^−^ (*m/z* 821.39784), pixel size of 35 μm, DHAP matrix. (F) Distribution of CFZ [M+H]^+^ (*m/z* 473.12942), pixel size of 35 μm. (G to J) Higher magnifications of selected regions from panel C (rectangles) for detection of AFB within the rim of macrophages (G) and the center of necrotic granulomas (H to J). The MALDI-MS imaging measurements of PZA and CFZ (shifted by 15 μm in *x* and *y* direction) and also immunohistochemical CD68/ZN staining were conducted on the same cryosection. Red arrows indicate cord formation. Scale bars, 1 mm (A to F); 10 μm (G to J).

By application of the established workflow, the impact of lesion heterogeneity on drug penetration was investigated in BALB/c IL-13^tg^ mice. To this end, therapy started 9 weeks after M. tuberculosis infection, mice were treated with the CFZ/PZA/RIF combination for 10 days, and 1 h after the last administration, lung tissue was collected and serial cryosections were prepared for histological characterization of lesions in combination with MALDI-MS imaging analysis. Since therapy started after necrotic pathology had developed, large granulomas consisting of necrotizing centers with karyorrhectic debris (Fig. 5A, n) that were surrounded by a collagen-rich fibrous layer (Fig. 5B) were readily apparent in addition to areas with cellular infiltration (Fig. 5A, c). A more detailed characterization of lesions by a combination of CD68 staining and Ziehl-Neelsen (ZN) staining revealed a rim of macrophages (Fig. 5C) on the inner side of the fibrotic capsule that were heavily infected with acid-fast rod-shaped M. tuberculosis (Fig. 5G). Besides these intracellular mycobacteria, high numbers of extracellular AFB were also observed within the necrotic center of granulomas (Fig. 5H to J), and here, even cording of mycobacteria, which is a characteristic feature of extracellular mycobacteria, was detected (Fig. 5H and I, red arrows).

MALDI-MS imaging analysis was performed in identical or neighboring cryosections to correlate the drug distribution with lesion pathology. The first-line drug PZA (Fig. 5D) exhibited a homogeneous distribution throughout the lung tissue and was detected within histopathologically normal lung tissue and areas with cellular infiltration as well as centrally necrotizing granulomas. The first-line drug RIF was present at high levels in uninvolved lung tissue and cellular areas, while lower signal intensities were detected within centrally necrotizing granulomas (Fig. 5E). The second-line drug CFZ was detectable within histopathologically normal lung tissue and in cellular inflammatory lesions, whereas only low intensities were measured within the core of large centrally necrotizing granulomas (Fig. 5F). Importantly, after the detection of PZA and CFZ (Fig. 5D and F) by MALDI-MS imaging, the same cryosection was reutilized for the histological characterization by CD68/ZN staining (Fig. 5C and G to J), allowing a direct comparison of drug distributions and target localizations. With this approach, we could clearly demonstrate that the first-line drug PZA penetrates tissue harboring intracellular and extracellular mycobacteria, while CFZ intensity is low in the center of large necrotizing granulomas, which, however, contains an enormous load of extracellular bacilli.

M. tuberculosis-infected BALB/c IL-13^tg^ mice develop heterogeneous pulmonary granulomas ranging from cellular inflammatory lesions through highly organized encapsulated granulomas with a cellular core to stratified centrally necrotizing granulomas (Fig. S2). Interestingly, depending on the lesion composition, CFZ abundance was higher in some cellular, inflammatory lesions (Fig. S2F), appeared to penetrate the rim of macrophages of smaller granulomas (Fig. S2F, stars), and was also detectable in the center of a stratified, encapsulated granuloma with a cellular core (Fig. S2A, encircled granuloma, and Fig. S2F). Regarding the distribution of the first-line drugs, PZA penetrated all lesion types (Fig. S2D), whereas the detection of RIF (Fig. S2E) was reduced in centrally necrotizing granulomas, confirming our observations described above.

While MALDI-MS imaging provides valuable information regarding the distribution of drugs within specific lesion compartments, absolute quantification of drugs is challenging. Therefore, drug concentrations within an entire neighboring cryosection were determined by LC-MS/MS measurements, yielding concentrations of 76 ng/mg for PZA, 25 ng/mg for CFZ, and 4 ng/mg for RIF.

**FIG 6:**
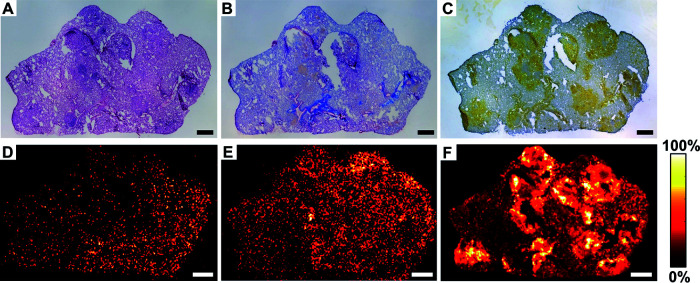
Distribution of CFZ, RIF, and PZA in cellular, inflammatory granulomas of BALB/c mice. BALB/c mice were infected with 263 CFU of M. tuberculosis H37Rv. After 9 weeks, animals were treated with CFZ/PZA/RIF for 10 days, and 1 h after the last administration, lung tissue was collected and serial cryosections were prepared for histological characterization of lesions and MALDI-MS imaging analysis. Correlation of lesion pathology (A to C) and drug distribution (D to F) in neighboring lung cryosections of a BALB/c mouse is shown. (A) HE staining revealed cellular, inflammatory lesions mainly consisting of macrophages detected by CD68/ZN staining (C) and clusters of lymphocytes but lacking a collagen encapsulation (B). (D) Distribution of PZA [M + 2H]^+·^ (*m/z* 125.05836), pixel size of 35 μm, DHB matrix. (E) Distribution of RIF [M-H]^−^ (*m/z* 821.39784), pixel size of 35 μm, DHAP matrix. (F) Distribution of CFZ [M+H]^+^ (*m/z* 473.12942), pixel size of 35 μm. The measurements of CFZ and RIF were conducted on the same cryosection (shifted by 15 μm in *x* and *y* direction). Scale bar, 0.5 mm.

As most preclinical efficacy studies for anti-TB drugs are performed in BALB/c mice, the pulmonary drug concentrations and distributions were also analyzed in BALB/c IL-13^tg^-negative littermates by applying the same treatment regimen and sample preparation as that described for BALB/c IL-13^tg^ mice. Again, neighboring cryosections were used for histological characterization of lesions and determination of drug distributions and concentrations. The histological evaluation of pulmonary lesions revealed cellular, inflammatory lesions (Fig. 6A; Fig. S3A) composed predominantly of CD68-positive macrophages (Fig. 6C; Fig. S3C) with aggregates of lymphocytes interspersed throughout the lesion. These less-well organized granulomas were not separated by a collagen rim from the adjoining tissue (Fig. 6B; Fig. S3B) and did not show signs of necrosis. The drug distribution within these lesions was detected by MALDI-MS imaging in neighboring cryosections. The two first-line drugs PZA (Fig. 6D; Fig. S3D) and RIF (Fig. 6E; Fig. S3E) readily distributed in the cellular lesions of BALB/c mice and exhibited a relatively homogeneous penetration throughout the healthy tissue. Interestingly, CFZ was also detected throughout the section (Fig. 6F; Fig. S3F), but higher signal intensities were measured in those areas that correspond to cellular granulomas, which are composed primarily of large numbers of macrophages. Furthermore, drug concentrations were measured in neighboring cryosections by LC-MS/MS, resulting in concentrations of 37 ng/mg for PZA, 74 ng/mg for CFZ, and 6 ng/mg for RIF. Hence, with respect to the investigated drug distribution and concentration, the most striking differences between necrotizing granulomas and cellular granulomas were observed for CFZ.

## DISCUSSION

The histopathologic hallmark of the host response to infection with M. tuberculosis is the development of the cellular granuloma and its subsequent degeneration and necrosis. While the granuloma is capable of containing and walling off the infecting pathogen, it also provides a survival niche for mycobacteria in postprimary TB (5, 28, 29). TB patients develop a wide spectrum of pathology, and the progression of disease results in a complex and dynamic lesion heterogeneity. However, the most prominent lesion type is the highly stratified necrotic granuloma with a fibrous capsule that separates from the adjoining tissue a central region of necrosis in which large numbers of mycobacteria reside.

In anti-TB therapy, the extent of antibiotic distribution not only depends on the lesion type but is also drug specific, which was reported in a landmark study by Prideaux et al. (17). By exploiting MALDI-MS imaging, the authors could demonstrate that the first-line drug PZA penetrated centrally necrotizing granulomas of TB patients whereas the second-line drug CFZ did not diffuse into necrotic foci. Since the penetration of antibiotics into centrally necrotizing granulomas is one of the driving factors for an effective chemotherapy, animal models developing a human-like pathology in conjunction with drug detection by techniques such as MALDI-MS imaging should be implemented in the preclinical evaluation of novel drugs or regimens.

However, research using M. tuberculosis is inherently complex, as all work must be carried out in BSL-3 laboratories. Due to the size and complexity of work conducted in these facilities, they are usually not equipped with most of the specific scientific instrumentation, so that samples must be transferred out of the BSL-3 laboratories and safely transported to partner sites. This process is challenging because of high biological safety requirements.

Gamma irradiation was used previously to eliminate M. tuberculosis within rabbit tissue for subsequent drug detection by MALDI-MS imaging (13, 30, 31). In the present study, we established a protocol for the inactivation of M. tuberculosis by gamma irradiation in samples from BALB/c IL-13^tg^ mice because they develop a TB patient-like pulmonary pathology upon M. tuberculosis infection (18, 22). In a first attempt, frozen lung tissue biopsy specimens containing macroscopically visible lesions were gamma irradiated with dosages ranging from 16.10 to 48.30 kGy without achieving complete inactivation of M. tuberculosis. Interestingly, viable mycobacteria were eliminated in pulmonary lesion tissue obtained from infected rabbits by delivering a dose of 3 megarads (30 kGy) in a ^60^Co irradiator with all three rods in a full-power position (7, 13). Radionuclide irradiators typically use the isotopes cesium-137 or cobalt-60 to create an irradiation field. While ^60^Co produces gamma rays with energies of 1.173 and 1.332 MeV and has a half-life of 5.27 years, ^137^Cs produces gamma rays with a lower photon energy of 0.662 MeV and has a longer half-life of 30.17 years. Since the penetration power of gamma rays into tissue (beam quality) depends on photon energy, a higher-energy photon radiation is more penetrating and the dose is deposited at increased depths (32). Hence, while the higher beam quality of ^60^Co is sufficient to inactivate viable M. tuberculosis throughout rabbit tissue, the lower photon energy produced by the ^137^Cs source used in the present study does not completely penetrate the mouse tissue biopsy specimens, so that viable M. tuberculosis was detected despite high irradiation doses. Therefore, in the next step, the depth of tissue was reduced by irradiating lung cryosections prepared from M. tuberculosis-infected BALB/c IL-13^tg^ mice. A dose of 5.85 kGy was identified as sufficient for the inactivation of M. tuberculosis H37Rv within cryosections containing encapsulated, centrally necrotizing granulomas. Importantly, even though we established a highly standardized protocol for the irradiation procedure, the elimination of M. tuberculosis was always subsequently confirmed for every irradiation run by assessing irradiated reference sections in the MGIT growth system. Also, it is worthy of note that the irradiation dose of 5.85 kGy is appropriate for M. tuberculosis inactivation in cryosections and may differ for other samples, such as M. tuberculosis grown in liquid broth, different organs collected from infected animals, or even lung homogenate generated from BALB/c IL-13^tg^ mice.

Gamma rays cause damage to bacteria either directly by inducing DNA single- and double-strand breaks or indirectly by interacting with water and producing reactive oxygen species (33–35). Even though cryosections were irradiated on dry ice to reduce the mobility of free radicals and minimize adverse side effects, the impact of gamma irradiation on tissue integrity, drug concentration, and distribution was thoroughly assessed in the present study. While a massive distortion of the lung tissue architecture after irradiation of decellularized whole mouse lungs was detected by HE staining in a previous study (36), we observed obvious irradiation-induced alterations neither in healthy tissue, in cellular granulomas, nor in complex structures such as collagen-encapsulated necrotizing granulomas. Furthermore, radiation-induced damage of collagen was reported by several studies (37), and radiation doses of 25 kGy or higher were shown to cause significant damage to the collagen polypeptide backbone, whereas less damage was observed at 10 kGy (38). In the present study, no major alterations of collagen-containing structures such as the fibrous capsule surrounding necrotizing granulomas were observed after radiation. Moreover, no irradiation-induced changes regarding the cellular composition as analyzed by CD68 expression of macrophages were detected. Hence, we determined an irradiation dose that is high enough to eliminate M. tuberculosis while preserving the integrity of lung tissue cryosections that contain encapsulated centrally necrotizing granulomas.

With respect to the impact of irradiation on the drug concentration, no major differences were observed for PZA or RIF. For the lipophilic antibiotic CFZ, a reduction by 32% was detected, which is probably due to the indirect effect of irradiation, that is, the production of reactive oxygen species. This observation is in accordance with a previous study, where CFZ was shown to be prone to degradation under oxidative stress conditions, resulting in 35.56% CFZ degradation (39). Importantly, regarding the distribution of CFZ, no irradiation-induced changes were observed by MALDI-MS imaging. Furthermore, as we described elsewhere (82), by applying the identical procedure, we also observed for the two first-line drugs PZA and RIF similar distributions within lung cryosections irrespective of irradiation. Even though gamma irradiation was previously reported for the inactivation of M. tuberculosis for subsequent LC/MS-MS or MALDI-MS imaging analyses (7, 13, 30, 31), this is the first study to our knowledge that comprehensively investigated the effect of gamma irradiation on tissue morphology, including cellular integrity, drug concentration, and drug distribution. Since the concentration of 1 out of 3 drugs was significantly altered after irradiation, we suggest that the impact of radiation should be assessed whenever it is applied during sample processing.

Taking these findings together, we successfully established a workflow for the inactivation of M. tuberculosis by gamma irradiation without affecting lung tissue integrity and drug distribution so that biological samples containing bacteria of a high biological safety level can be analyzed in conventional laboratories equipped with sophisticated scientific instrumentation.

By applying the workflow for elimination of M. tuberculosis, we determined drug concentrations in lung cryosections prepared from M. tuberculosis-infected mice treated with a CFZ/PZA/RIF combination for 10 consecutive days. Lung tissue was collected 1 h after the last administration, because the time to maximum concentration (*T*_max_) for PZA within lung tissue was reported to occur 1 h postdose in C3HeB/FeJ mice, which also develop centrally necrotizing granulomas after M. tuberculosis infection (40). In whole-lung samples prepared from C3HeB/FeJ mice, a maximum PZA concentration of 76.5 ng/mg was determined, which corresponds to the PZA concentration measured in pulmonary cryosections of BALB/c IL-13^tg^ mice (76 ng/mg). The pulmonary PZA concentration reported here for BALB/c mice (37 ng/mg) is reduced compared to the previously published concentration of 122.7 ng/mg, most likely because we collected lung tissue 1 h after the last dosing, and the *T*_max_ for PZA occurred at 0.5 h in BALB/c mice (40).

The highly lipophilic second-line drug CFZ has a prolonged half-life and accumulates in tissue so that in naive BALB/c mice a single oral dose (25 mg/kg) resulted in a pulmonary maximum concentration of drug (*C*_max_) of 0.76 ng/mg, which increased to 29.98 ng/mg after 4 weeks of administration (41–43). For M. tuberculosis-infected BALB/c mice, a CFZ concentration of approximately 40 ng/mg was measured in the lung after 2 weeks of treatment (43). Considering that large variations in pulmonary CFZ concentration have already been observed in BALB/c mice (44), the herein-reported drug concentrations within cryosections of M. tuberculosis-infected mice (25 ng/mg in BALB/c IL-13^tg^; 74 ng/mg in BALB/c) are in good accordance with previously published data.

For the first-line drug RIF, blood concentrations ranging from 9.5 to 13.52 μg/mL were detected in BALB/c or C3HeB/FeJ mice and concentrations in lungs were reported to mirror those in blood (45–47). The RIF concentrations within lung cryosections measured in the present study are slightly reduced, with 4 ng/mg for BALB/c and 6 ng/mg for BALB/c IL-13^tg^ mice. As the RIF concentration peaks in blood around 2 h after dosing (45, 46) and we collected lung tissue 1 h after the last administration, it is reasonable to assume that a considerable amount of RIF has not yet reached the lung, explaining lower pulmonary concentrations.

By combining MALDI-MS imaging, which provides a high-resolution mapping of relative drug intensities, with histopathological analyses, it was demonstrated that the spatial distribution of anti-TB drugs within human granulomas differs for various lesion types and is also drug specific. With respect to the broad range of antibiotic penetration, PZA, at one end of the spectrum, diffused rapidly into necrotic granulomas, whereas CFZ, at the other end of the spectrum, showed poor penetration into caseous centers of granulomas (17). Therefore, to validate BALB/c IL-13^tg^ mice as a preclinical model for the lesion-specific partitioning of drugs, the distribution of PZA, CFZ, and RIF was analyzed in lung cryosections by MALDI-MS imaging in conjunction with a comprehensive characterization of granulomas by histological and immunohistochemical staining.

The small, hydrophilic drug PZA distributed evenly throughout the lung tissue, including cellular and centrally necrotizing granulomas surrounded by a fibrous cuff. Consequently, neither the collagen encapsulation nor the absence of prominent vascularity within the necrotic center constitutes a barrier for PZA, so that this first-line drug reaches not only intracellular M. tuberculosis within foamy macrophages but also the large number of extracellular bacteria within the necrotic core. Importantly, the PZA distribution observed in BALB/c IL-13^tg^ mice reflects the penetration pattern reported for human TB lesions, rabbits, and C3HeB/FeJ mice (15, 17, 40).

Regarding the partitioning of RIF into granulomas by 1 h after the last administration, a homogeneous distribution into cellular granulomas and uninvolved lung tissue of BALB/c IL-13^tg^ and littermate controls was observed, whereas the penetration into the center of necrotic granulomas in BALB/c IL-13^tg^ mice was reduced. The distribution pattern regarding necrotic lesions is in accordance with the previously described penetration of RIF observed in C3HeB/FeJ mice (48). Furthermore, studies investigating the partitioning of RIF into rabbit lesions revealed a distribution in uninvolved lung tissue und cellular lesions but low penetration into necrotic lesions by 2 h after a single dose. Also following multiple administrations, at 2 h postdosing, only limited RIF penetration into the caseum was detected, while by 6 h and 12 h postdosing, the drug had fully penetrated necrotic lesions (16, 31). Notably, in TB patients treated with a single dose of RIF, only poor drug penetration into necrotic granulomas was observed at different time points after administration, whereas RIF was shown to accumulate in necrotic foci under steady-state conditions after administration of 180 dosages (17). In the present study, mice received only 10 doses of the CFZ/RIF/PZA combination, and the penetration pattern was analyzed 1 h after the last administration. Since the limited penetration of RIF into necrotic lesions of BALB/c IL-13^tg^ mice corresponds to the distribution pattern observed in rabbit and human lesions after a single administration, we assume for our study that the accumulation of RIF within the lung tissue was not yet at steady-state conditions to fully penetrate the center of necrotic granulomas of BALB/c IL-13^tg^ mice. Also, the time point of tissue collection might have been too early for the detection of RIF within the necrotic core.

The combination of lesion characterization and MALDI-MS imaging demonstrated the most striking granuloma- and compartment-specific partitioning for the second-line drug CFZ, which accumulated within cellular granulomas of BALB/c mice but poorly penetrated bacteria-rich necrotic centers of BALB/c IL-13^tg^ mice. The correlation with immunohistochemical detection of macrophages showed that CFZ accumulates within these immune cells, which is in line with previous studies reporting that CFZ is sequestered in macrophages (49, 50). Interestingly, lesions of BALB/c IL-13^tg^ mice revealed that CFZ also penetrated cellular granulomas that were surrounded by a collagen rim, indicating that the fibrotic wall *per se* is not a major barrier to drug penetration. Also depending on the granuloma composition, CFZ appeared to diffuse into the rim of foamy macrophages that harbor intracellular M. tuberculosis and surround the necrotic core of collagen-encapsulated lesions. However, in the present study, CFZ was imaged at a low spatial resolution (35-μm pixel size) so that an adequate correlation of immune cells and drug distribution was not possible. Elsewhere, we revealed, by high-resolution mapping of CFZ in centrally necrotizing lesions of BALB/c IL-13^tg^ mice in combination with macrophage detection, an accumulation of the drug within the rim of foamy macrophages (82). In striking contrast, CFZ failed to efficiently diffuse into the necrotic core of granulomas, which, however, is a niche for extracellular mycobacteria, as demonstrated by acid-fast staining. These observations imply that the distribution of CFZ depends on effective vascularization and the presence of immune cells within the granuloma. Most importantly, the CFZ distribution within necrotic granulomas of BALB/c IL-13^tg^ mice resembles its penetration observed in human necrotic lesions, where CFZ showed a clear partitioning between the cellular rim and the necrotic core of lesions with an accumulation in cellular layers compared to the necrotic caseum (17). Moreover, the herein-detected distribution patterns for CFZ, PZA, and RIF within lesions of BALB/c IL-13^tg^ mice were confirmed by another study conducted by us (82). Remarkably, the CFZ accumulation in cellular granulomas of BALB/c mice and the poor penetration into centrally necrotizing granulomas developing in BALB/c IL-13^tg^ mice provide an explanation for the striking discrepancy regarding its efficacy in C3HeB/FeJ and BALB/c mouse strains. While CFZ was highly effective in BALB/c mice, it was active in C3HeB/FeJ mice only when treatment started before the formation of centrally necrotizing granulomas, whereas only limited activity was observed under conditions of necrotic granulomas (51, 52).

Hence, the present study emphasizes that the evaluation of novel anti-TB drugs and the selection of new regimens should be supported by penetration analyses in animal models developing a human-like TB pathology to improve the predictive value of preclinical models, because owing to uneven drug distribution, a spatial and temporal window of monotherapy which could foster the emergence of drug resistance in selected niches might occur. By analyzing drug penetration patterns in animal models with a heterogeneity of TB lesions, new drug combinations can be designed that could minimize the development of drug resistance and accelerate cure (3). However, currently, not one animal model fully recapitulates the diversity of lesion types that are observed in TB patients (6, 53).

Regarding the rabbit model of TB, aerosol-infected outbred New Zealand White rabbits develop a spectrum of disease states and progression. The large size of lesions, including caseous and cavitary granulomas, makes them well suited for studying the impact of lesion types and sublesional compartments on drug distribution and concentration (7, 54–56). Even though the rabbit model is most appropriate for lesion-centric pharmacokinetic/pharmacodynamic (PK/PD) analyses, it is too expensive to be a workhorse for comprehensive preclinical drug efficacy studies, including the assessment of relapse (57).

The mouse model is the most widely used experimental model in TB drug development because of the easy handling and low housing costs of mice. While many commonly used mouse strains (BALB/c or C57BL/6) contain nonnecrotic cellular granulomas, C3HeB/FeJ mice are a versatile model, since they develop centrally necrotizing lesions after M. tuberculosis infection (19). In this mouse substrain following aerosol M. tuberculosis infection, three distinct types of lesions can be observed, which are encapsulated caseous necrotic lesions that closely resemble classical human TB granulomas (type I), rapidly progressive granulocytic lesions mainly composed of neutrophils (type II), and small cellular inflammatory lesions (type III). However, this heterogeneous distribution of lesion types, their differential responses to chemotherapy, and most importantly the high percentage of death (10% to 40%) between weeks 4 and 7 after infection remain major challenges of the C3HeB/FeJ mouse model of TB (58–60). The advantage of BALB/c IL-13^tg^ mice is that after M. tuberculosis infection, they develop similar forms of granuloma heterogeneity with consistent and reproducible progression toward centrally necrotizing lesions. A further value of the BALB/c IL-13^tg^ mouse model is that the distribution of drugs within necrotic lesions and drug efficacy studies can be conducted in the same experimental model of TB (18, 22). Additionally, the BALB/c genetic background allows for a direct comparison of drug distributions and efficacies between BALB/c IL-13^tg^ mice and the standard BALB/c mouse model.

While postprimary TB with centrally necrotizing pulmonary granulomas makes up a substantial proportion of TB patients to be treated with antibiotics, there is a not an insignificant number of immunocompromised M. tuberculosis-infected individuals. HIV/AIDS and the treatment of patients suffering from autoimmune inflammatory diseases, such as rheumatoid arthritis or Crohn`s disease, with tumor necrosis factor (TNF) antagonists are associated with an increased risk of reactivating TB (61–64). These patients require immediate treatment, but their granulomatous lesions may have a histopathological appearance different from that of centrally necrotizing granulomas in postprimary TB and thus also different properties regarding the penetration of antibiotics. Therefore, it is important to also evaluate the drug distribution in animal models reflecting immunocompromised TB patients coinfected with HIV or under treatment with TNF antagonists in comparison to the penetration into highly stratified centrally necrotizing granulomas of BALB/c IL-13^tg^ mice as analyzed in the present study. In T cell-depleted or in inbred A/J mice (65, 66), interstitial inflammation, confluent granuloma necrosis, neutrophilia, and disseminated AFB represent the pathogenesis in HIV- and TB-coinfected patients and, thus, may provide valuable models to analyze drug penetration under this specific clinical condition of TB. In mice lacking TNF or its receptor TNFp55, granulomas with confluent necrosis can be detected early after M. tuberculosis infection. These lesions are characterized by a loosely organized structure with exacerbated neutrophil and mononuclear inflammation and confluent necrosis containing an overwhelming amount of AFB (67–69). Also, M. tuberculosis-infected mice expressing the human ortholog instead of the murine TNF and treated with the clinically used TNF antagonists infliximab, adalimumab, or etanercept develop large, diffuse granulomas with disorganized cellular infiltrations and necrotic areas harboring high numbers of AFB (70). These data demonstrate that TNF is critical to maintain granuloma integrity. Furthermore, by exploiting a low-dose aerosol model of latent infection, a spontaneous and uncontrolled reactivation was observed in TNF-deficient mice, which emphasizes the relevance of TNF in containing M. tuberculosis infection (71). Hence, TNF depletion in M. tuberculosis-infected mice represents an additional model to investigate drug distribution by MALDI-MS imaging to mimic reactivation of TB in latently infected individuals under anti-inflammatory treatment.

In summary, we here present a general workflow for M. tuberculosis inactivation in lung cryosections containing centrally necrotizing granulomas, allowing in-depth studies of drug distribution and drug quantification. Overall, we propose that BALB/c IL-13^tg^ mice are an advanced preclinical model for the improved evaluation of novel drugs and new regimens, since the drug distribution within necrotic granulomas of these animals recapitulates their penetration in human lesions, as demonstrated by the partitioning of PZA and CFZ, both representing one end of the broad drug distribution spectrum observed in human TB granulomas. Importantly, by supporting the selection of new drug combinations with lesion distribution studies in animal models developing a human-like pathology, multidrug coverage in sublesional compartments can be ensured, which reduces the occurrence of potential monotherapy and development of drug resistance.

## MATERIALS AND METHODS

### Mice.

IL-13^tg^ mice expressing murine *il13* under the control of the human CD2 locus control region (72) [Tg(CD4-Il13)431Anjm] backcrossed to the BALB/c genetic background and littermates negative for the IL-13 transgene were bred under specific-pathogen-free conditions at the Christian-Albrechts-University of Kiel (Kiel, Germany) or the Max-Planck-Institute for Evolutionary Anthropology (Leipzig, Germany). M. tuberculosis-infected mice were maintained in individually ventilated cages (IVC; Ebeco, Castrop-Rauxel, Germany) under BSL-3 conditions at the Research Center Borstel. Female BALB/c mice (Charles River Laboratories) receiving only drug treatment were housed under standard conditions at the Research Center Borstel.

### Bacteria and drugs.

M. tuberculosis H37Rv was grown in Middlebrook 7H9 broth (Difco, Detroit, MI, USA) supplemented with 10% oleic acid-albumin-dextrose-catalase (OADC; Life Technologies, Gaithersburg, MD, USA), 0.05% Tween 80, and 0.2% glycerol (VWR International GmbH, Darmstadt, Germany). Mid-exponential-phase cultures were harvested, aliquoted, and frozen at −80°C. After thawing, the bacterial suspension was drawn through a nonpyrogenic needle (Microlance3; BD, Drogheda, Ireland) to ensure proper dispersion of mycobacteria prior to aerosol infection. PZA, CFZ, and RIF were purchased from Sigma-Aldrich (Taufkirchen, Germany). RIF was ground to a small particle size before sterile water was added (73). PZA was dissolved in sterile water and incubated at 55°C until particles were dissolved. CFZ was prepared by grinding with a mortar and pestle and added to 0.05% (wt/vol) agarose dissolved in sterile water (51). Concentrated stocks of individual drugs were kept at 4°C and combined on the day of application. PZA solutions, if particulate, were heated to 55°C before combination. Daily drug dosages of 150 mg/kg for PZA, 25 mg/kg for CFZ, and 10 mg/kg for RIF were chosen to achieve human-equivalent doses (44).

### Aerosol infection and drug treatment.

Mice were exposed to a low- or intermediate-dose aerosol infection with the M. tuberculosis H37Rv strain in a Glas-Col aerosol chamber, as previously described (74). Inoculum size was confirmed 24 h after infection by determining the bacterial burden in the lung. Treatment was initiated 9 weeks after infection, and the CFZ/PZA/RIF drug combination was administered by oral gavage for 10 consecutive days. At 1 h after the last administration, the mice were euthanized by CO_2_ inhalation, lungs were aseptically removed, and lung tissue containing macroscopically visible lesions was snap-frozen in liquid nitrogen. BALB/c mice receiving only therapy without prior infection were treated with the CFZ/PZA/RIF combination by oral gavage for 5 consecutive days. Mice were sacrificed 0.5 h or 1 h after the last administration, and individual lung lobes were isolated and snap-frozen in liquid nitrogen. All animal experimentation was in accordance with the German Animal Protection Law and was approved by the animal research ethics committee of the federal state of Schleswig-Holstein (Germany) prior to permission by the Ministry of Energy, Agriculture, the Environment, Nature and Digitalization (Kiel, Germany; permit no. 3-1/15 and 69-6/16).

### Tissue cryosectioning.

Serial cryosections (12 to 14 μm) were cut from unembedded lung tissue at −25°C using a Leica CM1850 or CM3050s cryostat (Leica Microsystems, Wetzlar, Germany) and thaw mounted onto adhesive glass slides (SuperFrost) as described by Treu et al. (12). Sections were stored at −80°C until further processing.

### Gamma irradiation for inactivation of M. tuberculosis.

Further analysis of M. tuberculosis-containing samples required transfer to laboratories operating without biosafety level so that the inactivation of H37Rv by gamma irradiation had to be established. To this end, either lung tissue biopsy specimens or pulmonary cryosections frozen on dry ice were put into the irradiation chamber of a Biobeam 8000 (Gamma-Service Medical GmbH, Leipzig, Germany). The instrument is equipped with a ^137^Cs gamma ray source (gamma energy = 0.662 MeV) with a half-life of 30.17 years and an activity of 81.21 TBq. Radiation doses were measured using alanine dosimetry. Elimination of M. tuberculosis was assessed by incubating the irradiated samples for at least 6 weeks in MGIT tubes (Becton, Dickinson GmbH, Heidelberg, Germany) at the National Reference Center for Mycobacteria at the Research Center Borstel. The MGIT contain 7 mL of modified Middlebrook 7H9 broth base for the cultivation of almost all mycobacteria, including M. tuberculosis. The complete medium contains OADC enrichment to optimize the growth of mycobacteria. Any positive sample was examined for AFB and cord formation.

### Histology and immunohistochemistry.

To examine the development of centrally necrotizing granulomas, BALB/c IL-13^tg^ mice were sacrificed 9 weeks after infection, lung lobes were fixed in 4% formalin–phosphate-buffered saline (PBS), set in paraffin blocks, and sectioned (2 to 3 μm). Lung cryosections prepared from drug-treated BALB/c, BALB/c IL-13^tg^, or BALB/c IL-13^tg^-negative littermates were fixed in paraformaldehyde (PFA; 4% in PBS) (Morphisto, Frankfurt, Germany) or methanol. If MALDI-MS imaging analyses were performed prior to the immunohistochemical staining, the matrix was removed from the surface of the cryosection by gentle shaking in methanol. Standard protocols for HE and Heidenhain’s azan trichrome staining were used for histopathological analyses of paraffin-embedded sections or cryosections. For immunostaining, formalin-fixed and paraffin-embedded samples were deparaffinized, rehydrated, and subjected to a heat-induced epitope retrieval in the presence of 10 mM citric acid (pH 6.0). After cooling, slides were washed with TBS (50 mM Tris saline buffer, pH 7.6), and endogenous peroxidase activity was blocked by incubation with 3% H_2_O_2_ for 10 min. Following washing, sections were incubated in antibody diluent (Zytomed Systems GmbH, Berlin, Germany) containing a primary antibody specific for CD68 (ab125212; Abcam, Cambridge, UK) at room temperature with gentle agitation. After washing, a horseradish peroxidase (HRP)-conjugated polymer-based detection system (ZytoChem-Plus kit anti-rabbit; Zytomed Systems GmbH, Berlin, Germany) was used, followed by visualization with the chromogen 3-amino-9-ethylcarbazole (AEC; Permanent AEC kit; Zytomed Systems GmbH, Berlin, Germany), both according to the manufacturer’s instructions. Sections were counterstained with Gill’s hematoxylin, dehydrated, and coverslipped with Pertex (Medite, Burgdorf, Germany) mounting medium. To visualize M. tuberculosis and CD68 in the same lung section, fixed cryosections were stained for AFB using a ZN carbol-fuchsin solution (Merck, Darmstadt, Germany). After washing, endogenous peroxidases and biotin were blocked, followed by incubation with primary antibody against CD68 (ab125212 or ab53444, clone FA-11; Abcam, Cambridge, UK). For detection, biotinylated goat anti-rabbit antibody (Jackson Immunoresearch, Suffolk, UK) or biotinylated rabbit anti-rat IgG (Vector Laboratories, Burlingame, CA, USA), followed by the Vectastain Elite ABC peroxidase kit (Vector Laboratories, Burlingame, CA, USA), was used. Peroxidase was revealed by StayYellow/HRP (Abcam, Cambridge, UK) as the chromogen, and sections were counterstained with methylene blue (Merck, Darmstadt, Germany). Slides were imaged using a BX41 light microscope (Olympus, Hamburg, Germany) and NIS-Elements software (Nikon, Badhoevedorp, The Netherlands). For whole sections, images were stitched with Image Composite Editor (Microsoft, Redmond, WA, USA).

### MALDI-MS imaging analysis.

Lung cryosections were shipped frozen from the Research Center Borstel to the University of Bayreuth for MALDI-MS imaging analysis and stored at −80°C. Tissue sections were transferred into a desiccator for 10 min prior to matrix application by using a semiautomatic pneumatic sprayer system. Matrix solutions were prepared as follows: 2,5-dihydroxybenzoic acid (DHB), 20 mg/mL (Sigma-Aldrich, Taufkirchen, Germany), or 2,6-dihydroxyacetophenone (DHAP) (Sigma-Aldrich, Taufkirchen, Germany), 10 mg/mL, in methanol (MeOH)-H_2_O (1:1, vol/vol). DHB was acidified with 0.1% (vol/vol) trifluoroacetic acid. MALDI-MS imaging measurements were performed using an AP-SMALDI10 source (TransMIT GmbH, Gießen, Germany) equipped with a 337-nm (λ) N_2_ laser operating at a repetition rate of 60 Hz, coupled to a Q Exactive HF (Thermo Fisher Scientific GmbH, Bremen, Germany) orbital trapping mass spectrometer (75, 76). Measurements were carried out in positive and negative ion modes with one scanning event and 30 shots per pixel at a mass resolution of 240,000 at *m/z* 200 full width at half maximum. All measurements were performed with a fixed C-trap injection time of 500 ms. Matrix clusters of known sum formula (77) and the deprotonated molecule of phosphatidylserine (38:4) (C_44_H_77_NO_10_P^−^) were used for internal mass calibration. Conversion of proprietary Thermo RAW files to the open data format imzML (78) was performed using the Java-based open access software jimzMLConverter version 1.3 (79). Data visualization was performed using MSiReader version 1.0 (80) with a bin width of ±2.5 ppm. Extracted ion images were interpolated using the linear interpolation setting 2. Mass-to-charge ratios of drug compounds are given as the calculated theoretical mass. Mass measurement accuracies are given as the root mean square error (RMSE) of the δ*m/z* values in parts per million of each individual spectrum containing the targeted ion within a ±2.5 ppm window of the theoretical mass in Table S1 in the supplemental material. Compound identification was based on accurate mass. To confirm the identification of the drugs, lung sections of BALB/c IL-13^tg^ mice, which were not dosed with the drug combination (CFZ/PZA/RIF), were analyzed as negative control samples as we described elsewhere (82).

### Extraction procedure for lung cryosections.

Prior to preparation for LC-MS/MS measurements, cryosections were photographed to determine the area of the sections using Fiji ImageJ software. Based on the known thickness of the cryosections and by assuming a density of 1 g/cm^3^, the weights of the cryosections were estimated. To measure the drug concentration, the sections were detached from the glass slides by suspending them in sterile water and subsequently dried using a SpeedVac. Afterwards, samples were reconstituted with 25 μL LC-MS-grade water, 200 μL acetonitrile (ACN) containing 12.5 ng/mL reserpine, and 25 μL 1% formic acid (FA). Samples were incubated at room temperature for 10 min under constant shaking and afterwards centrifuged for 10 min at 15,000 × *g* at room temperature. Approximately 200 μL of the resulting supernatant was transferred to a 1.5-mL Eppendorf tube and recentrifuged under the same conditions. Afterwards, 50 μL supernatant was transferred in a vial (three aliquots per sample), and the injection volume was 5 μL for the LC-MS/MS analysis.

### Extraction procedure for lung homogenates.

Lung homogenate samples from untreated mice, prepared as previously described (81), were dried in a SpeedVac and subsequently reconstituted in 100 μL LC-MS-grade water, 800 μL ACN, and 100 μL 1% FA. The mixture was incubated at room temperature for 10 min under constant shaking, before the samples were centrifuged for 10 min at 15,000 × *g.* The resulting supernatant, approximately 700 μL, was collected in a separate tube and centrifuged under the same conditions to avoid floating particles. As a reference for the quantification, reserpine solution (12.5 ng/mL in ACN) was added to all samples. Calibration standards were prepared by diluting stock solutions in lung tissue homogenate from naive mice with individual standard curve ranges of 0.001 to 0.25 μg/mL for CFZ, 0.001 to 1 μg/mL for PZA, and 0.0025 to 0.1 μg/mL for RIF.

### Quantitation of antibiotics by LC-MS/MS.

LC-MS/MS analysis was performed using a Waters Micromass Quattro Premier XE triple-quadrupole mass spectrometer (Waters Corporation, Milford, MA, USA) coupled to an 1100 series high-performance liquid chromatography (HPLC) system (Agilent Technologies, Santa Clara, CA, USA) using electrospray ionization (ESI). Samples were kept at 4°C in the autosampler during batch acquisitions.

For LC separation, a SeQuant ZIC-HILIC column (Merck Millipore SeQuant; 2.1-mm inner diameter by 150-mm length with a 5-μm particle size and a pore size of 200 Å) at a column temperature of 30°C was used. One percent FA (solvent A) and ACN (solvent B) were used as the mobile phase. The gradient started at 90% B at a flow rate of 0.5 mL/min. After 1 min of isocratic conditions, the percentage of ACN was decreased to 2% B until minute four. At minute four, the flow rate was increased to 0.8 mL/min. The gradient was kept isocratic at 2% B with a flow rate of 0.8 mL/min for 6 min until minute 10. Afterwards, the percentage of ACN was reincreased to 90% B until minute 15 and the flow rate was redecreased to 0.5 mL/min after minute 19. The conditions were maintained for 1 min, so that the total run time was 20 min (Table S2).

Multiple reaction monitoring (MRM) was used to quantify all analytes, and the corresponding transitions are listed in Table S3. Electrospray ionization in the positive ion mode was utilized with the following source parameters. The cone gas flow and desolvation gas flow were set to 100 L/h and 800 L/h, respectively. The extractor voltage was 3.0 V. The capillary voltage was set to 3.0 kV, the source temperature to 90°C, and the desolvation gas temperature to 450°C. MassLynx 4.1 (Waters Corporation, Milford, MA, USA) was used for operating the platform, and TargetLynx was used for data analysis. Quantitation was based on external calibration using untreated murine lung tissue in reference to reserpine.

### Statistical analysis.

If applicable, statistical analysis was performed using Prism 8 (GraphPad Software, San Diego, CA, USA). Quantifiable data are expressed as the means of individual determinations and standard deviations (SD). For determining the recovery rates after irradiation for PZA, CFZ, or RIF, nonirradiated and irradiated cryosections were compared using the Wilcoxon signed rank test.
