# Supplementary material for: Interleukin-13-Overexpressing Mice Represent an Advanced Preclinical Model for Detecting the Distribution of Antimycobacterial Drugs within Centrally Necrotizing Granulomas
Source: Antimicrob Agents Chemother. 2022 May 23;66(6):e01588-21. doi: 10.1128/aac.01588-21 (PMC9211424; doi:10.1128/aac.01588-21)
Supplement: Supplemental file 1 — Supplemental material. Download aac.01588-21-s0001.pdf, PDF file, 5.9 MB [file aac.01588-21-s0001.pdf]

Interleukin-13 overexpressing mice represent an advanced pre-clinical model for detecting the distribution of anti-mycobacterial drugs within centrally necrotizing granulomas

Kerstin Walter, Julia Kokesch-Himmelreich, Axel Treu, Franziska Waldow, Doris Hillemann, Nikolas Jakobs, Ann-Kathrin Lemm, Dominik Schwudke, Andreas Römpp, and Christoph Hölscher

SUPPLEMENTAL MATERIAL

TABLE S1. Calculated RMSE values (ppm) for all drug compounds in the MS imaging experiments.

The figure of the corresponding MS image is given in the first column. The theoretical mass of the analyte is shown in the third column. The number of spectra in which the analyte was detected is given in the fourth column. The root mean square error (RMSE) of the Δm values in ppm of all spectra containing the targeted ion was calculated (last column).

Figure	Analyte	Theoretical *m/z*	Number of spectra	RMSE value / ppm
4B	CFZ $[M+H]^+$	473.12942	29704	1.5
5D	PZA $[M+2H]^{+\cdot}$	125.05836	3132	0.43
5E	RIF $[M-H]^-$	821.39784	5229	0.68
5F	CFZ $[M+H]^+$	473.12942	14483	0.68
S2 D	PZA $[M+2H]^{+\cdot}$	125.05836	4319	0.81
S2 E	RIF $[M-H]^-$	821.39784	4319	0.81
S2 F	CFZ $[M+H]^+$	473.12942	25658	0.44
6D	PZA $[M+2H]^{+\cdot}$	125.05836	2781	0.97
6E	RIF $[M-H]^-$	821.39784	5159	1.3
6F	CFZ $[M+H]^+$	473.12942	19213	0.56
S3 D	PZA $[M+2H]^{+\cdot}$	125.05836	1815	0.43
S3 E	RIF $[M-H]^-$	821.39784	2774	0.82
S3 F	CFZ $[M+H]^+$	473.12942	6935	0.38

TABLE S2. Conditions of the hydrophilic interaction liquid chromatography.

Solvent A: 1% formic acid. Solvent B: acetonitril.

Time [min]	B [%]	Flow [mL/min]
0.0	90.0	0.5
1.0	90.0	0.5
4.0	2.0	0.5
4.1	2.0	0.8
10.0	2.0	0.8
15.0	90.0	0.8
19.0	90.0	0.8
20.0	90.0	0.5

TABLE S3. Mass spectrometry conditions including transition of CFZ, PZA, RIF and reserpine (used as internal standard).

Analyte	Parent [*m/z*]	Daughter [*m/z*]	Cone Voltage [EV]	Collision Energy [EV]	Dwell [s]
clofazimine	471.6	395.3	60	45	0.05
pyrazinamide	123.7	78.9	20	20	0.05
rifampicin	823.5	791.0	30	20	0.05
**Internal standard**:					
reserpine	608.6	194.9	30	35	0.05

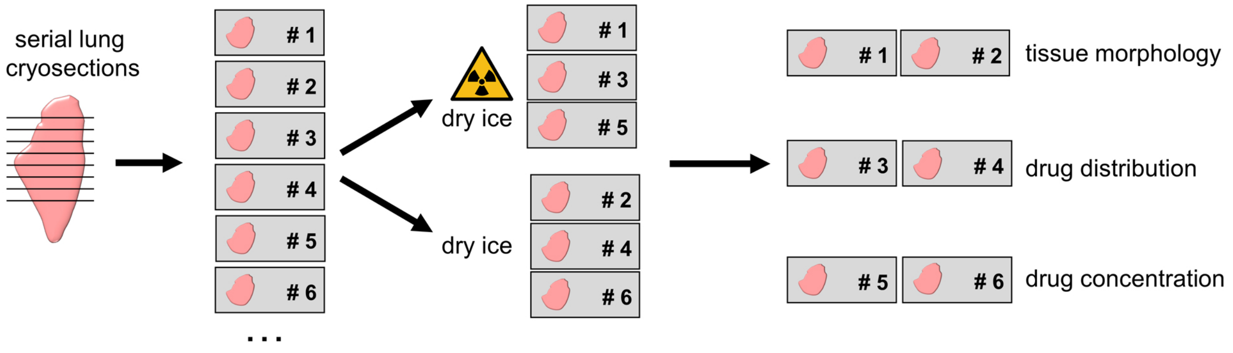

FIG. S1. Schematic illustration of sample preparation to assess the impact of gamma irradiation on tissue morphology, drug distribution and drug concentration.

Serial cryosections were cut from lungs of CFZ/PZA/RIF treated either Mtb-infected BALB/c IL-13^{tg} mice or naïve BALB/c mice. Every other cryosection was gamma-irradiated with a dose of 5.85 kGy on dry ice while the adjacent cryosections were cooled on dry ice during the irradiation process. The effect of irradiation was investigated by comparing the tissue morphology, drug distribution and drug concentration in directly neighboring cryosections that were either irradiated or non-irradiated.

TABLE S4. Drug concentrations in irradiated and non-irradiated cryosections and calculation of the recovery rate.

Lungs were harvested from naïve BALB/c mice treated with the CFZ/PZA/RIF drug combination. Serial cryosections were prepared and every other section was irradiated before samples were processed for LC-MS/MS measurements. Drug concentrations were determined in 2 mice (#1 and #2) and cryosections of mouse #1 were irradiated, processed and measured at two different time points and are therefore referred to as 1. batch and 2. batch. The mean drug concentrations were calculated for those cryosections that were not irradiated and the drug concentrations in every irradiated cryosection were related to these mean values resulting in the recovery rate plotted in Fig. 4A.

**drug**	**mouse**	**drug concentration [ng/mg]**		**recovery rate [%]**
		**w/o irradiation**	**with irradiation**	
PZA	**#1** 1. batch	104.16	98.76	**84**
		128.46	149.00	**126**
		118.87	183.46	**155**
		118.43	110.60	**94**
		97.13	125.11	**106**
		142.60	152.09	**129**
	**mean**	**118.28**		
	**#1** 2. batch	41.38	40.71	**101**
		37.12	50.79	**126**
		34.10	29.85	**74**
		50.64	45.13	**112**
		39.00	32.17	**80**
	**mean**	**40.45**		
	**#2**	55.78	61.70	**106**
		63.47	66.89	**115**
		44.45	42.98	**74**
		64.15	54.19	**93**
		75.92	64.05	**110**
		42.18	29.50	**51**
		50.90	45.77	**79**
		58.43	59.05	**102**
		70.89	58.84	**101**
		54.28	60.48	**104**
	**mean**	**58.05**		

drug	mouse	drug concentration [ng/mg]		recovery rate [%]
		w/o irradiation	with irradiation	
RIF	**#1** 1. batch	8.41	7.37	**68**
		11.01	10.63	**99**
		11.82	12.49	**116**
		14.91	11.19	**104**
		8.77	12.69	**118**
		9.76	11.11	**103**
	**mean**	**10.78**		
	**#1** 2. batch	6.31	6.86	**138**
		4.15	6.09	**123**
		3.79	3.54	**71**
		6.26	4.98	**100**
		4.31	3.33	**67**
	**mean**	**4.96**		
	**#2**	1.64	1.38	**117**
		1.34	1.29	**110**
		0.84	0.83	**71**
		1.18	0.90	**76**
		1.29	0.97	**82**
		0.75	0.83	**71**
		1.15	0.93	**79**
		1.11	1.40	**119**
		1.44	1.03	**88**
		1.03	0.68	**58**
	**mean**	**1.18**		

drug	mouse	drug concentration [ng/mg]		recovery rate [%]
		w/o irradiation	with irradiation	
CFZ	**#1** 1. batch	15.21	10.94	**50**
		21.49	14.04	**64**
		23.24	12.97	**59**
		23.54	14.34	**65**
		21.08	10.75	**49**
		27.49	12.76	**58**
	**mean**	**22.01**		
	**#1** 2. batch	12.49	8.17	**78**
		8.11	9.65	**92**
		7.48	5.50	**52**
		14.42	8.01	**76**
		9.89	5.43	**52**
	**mean**	**10.48**		
	**#2**	18.81	16.80	**90**
		16.01	16.20	**87**
		14.70	13.89	**74**
		23.60	11.89	**64**
		26.27	16.26	**87**
		16.64	5.99	**32**
		18.36	14.91	**80**
		7.81	19.24	**103**
		19.92	10.37	**55**
		24.83	13.15	**70**
	**mean**	**18.70**		

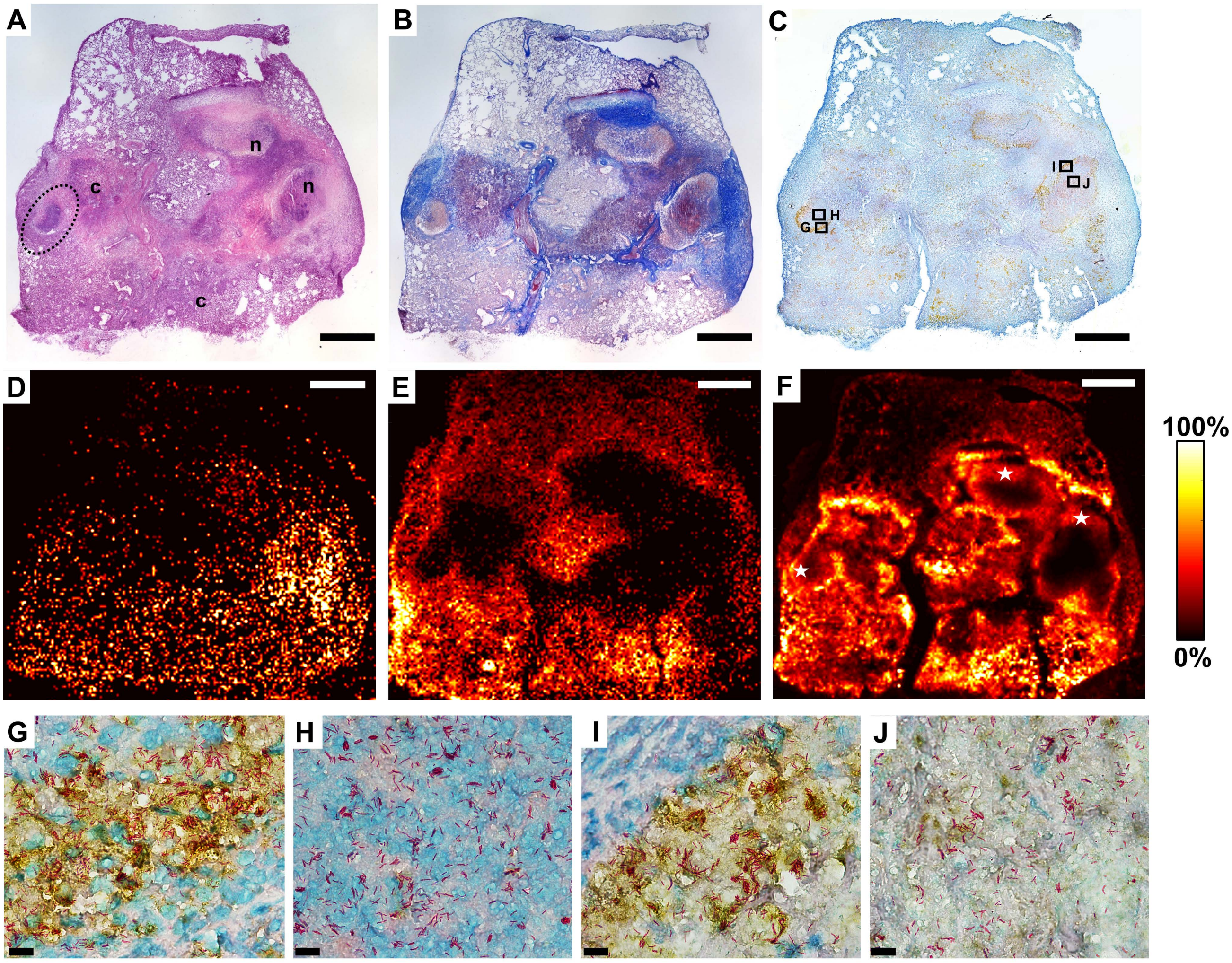

FIG. S2. Distribution of CFZ, RIF and PZA in centrally necrotizing granulomas of BALB/c IL-13^{tg} mice.

BALB/c IL-13^{tg} mice were infected with 263 CFU of Mtb H37Rv. After 9 weeks, animals were treated with CFZ/PZA/RIF for 10 days and 1 h after the last administration lung tissue was collected and serial cryosections were prepared for histological characterization of lesions and MALDI MS imaging analysis. Correlation of lesion pathology **(A-C; G-J)** and drug distribution **(D-F)** in neighboring lung cryosections of a second BALB/c IL-13^{tg} mouse. **(A)** HE staining revealed cellular, inflammatory lesions (c), and centrally necrotizing granulomas (n). A highly organized granuloma with a collagen capsule next to a rim of macrophages and a cellular core is encircled by a dotted line. Stratified granulomas are surrounded by a rim of macrophages **(C)** detected by CD68/ZN staining and a collagen capsule (**B**) detected by trichrome staining. **(D)** Distribution of PZA $[M+2H]^{+\cdot}$ (*m/z* 125.05836), pixel size: 40 µm, DHB matrix. **(E)** Distribution of RIF $[M-H]^{-}$ (*m/z* 821.39784), pixel size: 40 µm, DHAP matrix. **(F)** Distribution of CFZ $[M+H]^{+}$ (*m/z* 473.12942), pixel size: 40 µm, ★ CFZ detection within encapsulated granulomas. (**G-J)** Higher magnifications of selected regions in C (rectangles) for detection of AFB within the rim of macrophages (G, I) and center of structured cellular (H) or necrotic granulomas (J). The MALDI MS imaging measurements of RIF and CFZ (shifted by 20 µm in x and y direction) and also immunohistochemical CD68/ZN staining were conducted on the same cryosection. Scale bar A-F: 1 mm; G-J: 10 µm.

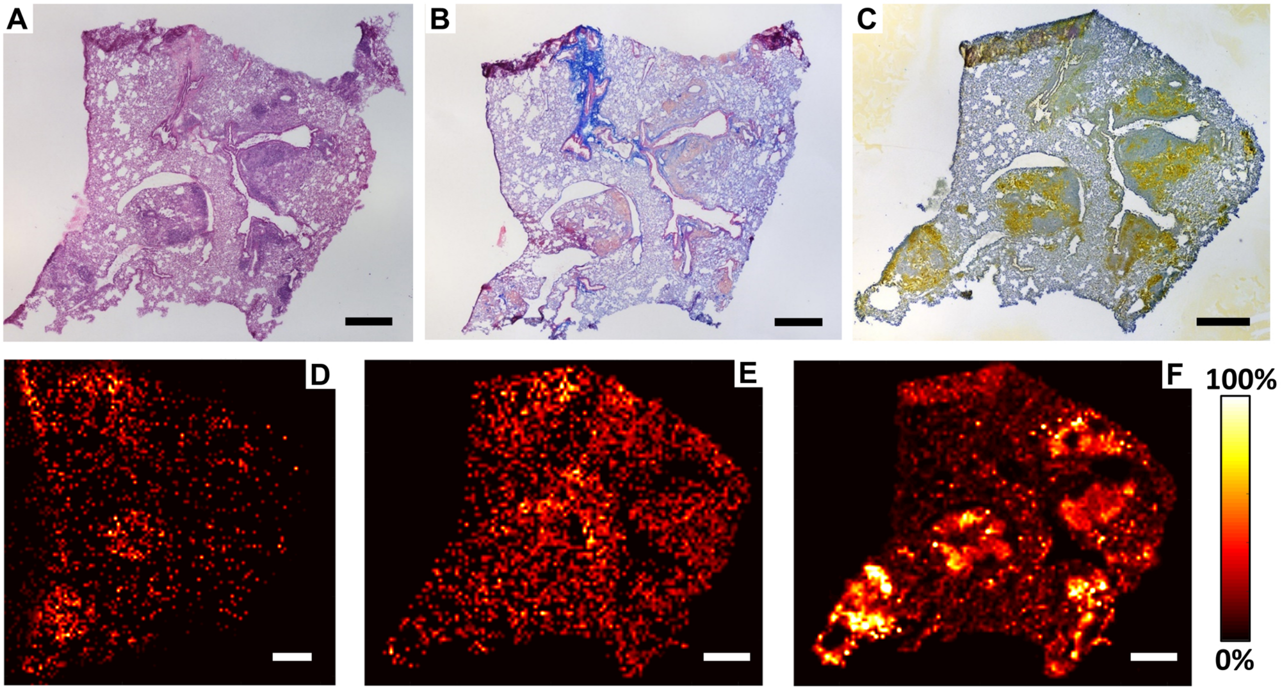

FIG. S3. Distribution of CFZ, RIF and PZA in cellular, inflammatory granulomas of BALB/c mice.

BALB/c mice were infected with 263 CFU of Mtb H37Rv. After 9 weeks, animals were treated with CFZ/PZA/RIF for 10 days and 1 h after the last administration lung tissue was collected and serial cryosections were prepared for histological characterization of lesions and MALDI MS imaging analysis. Correlation of lesion pathology **(A-C)** and drug distribution **(D-F)** in neighboring lung cryosections of a BALB/c mouse. **(A)** HE staining revealed cellular, inflammatory lesions mainly consisting of macrophages as detected by CD68/ZN staining **(C)** and clusters of lymphocytes but lack a collagen encapsulation **(B)**. **(D)** Distribution of PZA $[M+2H]^{+\cdot}$ (*m/z* 125.05836), pixel size: 35 µm, DHB matrix. **(E)** Distribution of RIF $[M-H]^{-}$ (*m/z* 821.39784), pixel size: 35 µm, DHAP matrix. **(F)** Distribution of CFZ $[M+H]^{+}$ (*m/z* 473.12942), pixel size: 35 µm. The measurements of CFZ and RIF were conducted on the same cryosection (shifted by 15 µm in x and y direction). Scale bar: 0.5 mm.
